# Retrospective Evaluation of Clinical and Radiological Results of Patients Treated With Arthroscopic Transosseous Repair for Rotator Cuff Tear

**DOI:** 10.7759/cureus.71004

**Published:** 2024-10-07

**Authors:** Mustafa Aydın, Ahmet Fırat

**Affiliations:** 1 Orthopedics and Traumatology, Gülhane Training and Research Hospital, Ankara, TUR; 2 Orthopedics and Traumatology, Etlik City Hospital, Ankara, TUR

**Keywords:** ato technique, chronic joint pain, cortical implant, ortho-biologics, rotator cuff tears

## Abstract

Background: Surgical repair is recommended for symptomatic full-thickness rotator cuff tears to restore muscle function and alleviate pain. Advances in arthroscopic techniques and new implant designs have led to more reliable repair methods. The choice of technique is crucial for achieving favorable clinical outcomes.

Objectives: This study evaluates the short-term clinical and radiological outcomes of patients treated with the arthroscopic transosseous technique (ATO).

Methods: Data from 43 patients who underwent full-thickness rotator cuff (RC) repair using the arthroscopic transosseous technique between February 2014 and April 2016 were prospectively collected and retrospectively reviewed. Included patients had medium-sized (1-3 cm) full-thickness supraspinatus tears extending to the infraspinatus and underwent tenotomy or biceps tenodesis. Functional outcomes were assessed using the Visual Analog Scale (VAS), American Shoulder and Elbow Surgeons (ASES) score, and constant score at their final follow-up appointment.

Result: Radiological evaluation included postoperative MRI to assess re-tear development. The mean follow-up period was 26.52±8.14 months. Postoperative VAS, ASES, and constant scores significantly improved compared to preoperative values (3.14±1.20, 88.4±8.12, and 88.9±10.6, respectively).

Conclusion: The ATO technique provides strong mechanical and biological repair, yielding good functional outcomes for full-thickness rotator cuff tears (RCTs). It is an effective method for early recovery of shoulder joint range of motion (ROM) and pain reduction.

## Introduction

The prevalence of rotator cuff tears (RCT) significantly increases with age and is a common cause of shoulder joint pain and restricted movement [1]. Surgical repair is preferred for symptomatic RCTs to reduce pain and restore muscle function. The technique used in the repair is critical for achieving satisfactory clinical outcomes [2,3].

Single-row (SR) repair methods have been reported to fail in restoring the rotator cuff (RC) attachment site, which is known as footprint, thus leading to high failure rates [4,5]. Advances in arthroscopic techniques and implant designs have introduced safer and more reliable fixation options like double row (DR) and arthroscopic transosseous equivalent (TOE) suture bridge techniques, as well as transosseous techniques (ATO) with or without implants. These developments aim to increase contact area at the footprint, provide stable repair under tensile loads, and improve bone-tendon healing and functional outcomes.

The most common postoperative complication is re-tearing due to insufficient repair [6]. Failures in RCT repair are often caused by knot failure, suture loosening, implant pullout, and anchor fixation loss [7]. Reports indicate that anchor fixation loss and implant pullout result in 10% to 80% of revision RC repair surgeries [8]. Anchor fixation strength is influenced by bone density, anchor design, and repair technique [9]. Transosseous sutures have been reported to provide greater contact area than anchor repairs [10]. The aim of the study is to evaluate the short-term clinical and radiological outcomes of patients who underwent RCT repair with the ATO technique retrospectively.

## Materials and methods

Ethical approval and informed consent were obtained from all patients. Data from 43 patients who underwent full-thickness RCT repair using the ATO technique between February 2014 and April 2016 were prospectively collected and retrospectively reviewed. All patients were operated on by a single experienced arthroscopic surgeon.

The inclusion criteria for the study were determined as having a medium (1-3 cm) full-thickness supraspinatus tear extending to the infraspinatus or supraspinatus according to Cofield classification [11], having undergone RCT repair with ATO technique, including tenotomy or biceps tenodesis, and having attended postoperative check-ups regularly within the first year.

Patients who had subscapularis tear, Goutallier stage 3-4 fatty infiltration, frozen shoulder, humeral head osteonecrosis or osteomyelitis, shoulder joint arthritis, and those who had undergone arthroscopic repair of a superior labrum anterior-posterior (SLAP) lesion or any other surgery on the same shoulder previously were excluded from the study. Thirty-three patients were included in the study according to these criteria.

Surgical technique

**Figure 1:**
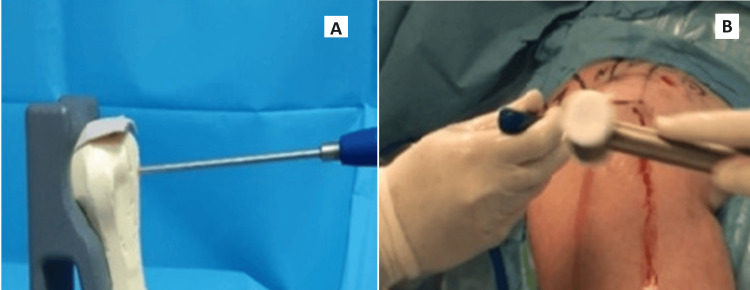
(A) Picture demonstrate opening an entry hole for a transosseous implant; (B) picture of opening an entry hole for a transosseous implant intraoperatively

All surgeries were performed under general anesthesia in the beach chair position. Diagnostic arthroscopy confirmed the joint. After bursectomy and acromioplasty, the footprint and tear edges were refreshed. A tunnel was drilled 18-20 mm distal to the lateral tubercle corner, aligned with the tear center (Figure 1).

**Figure 2:**
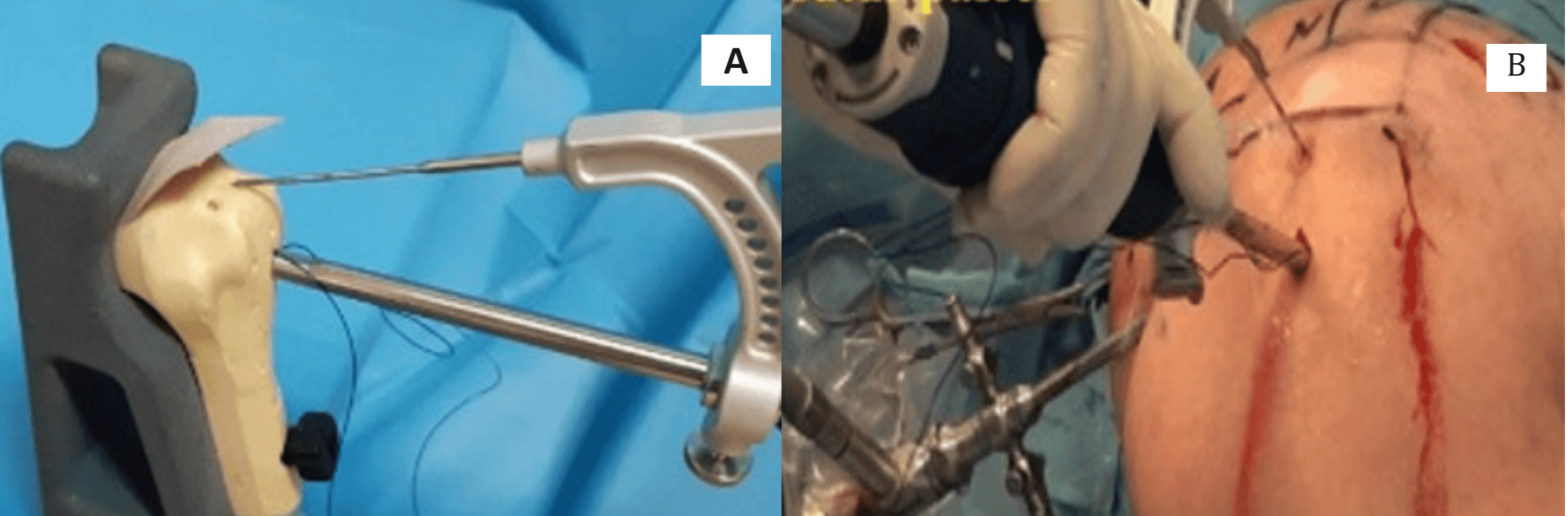
(A) Demonstrate of placement the transosseous suture passer with the targeter; (B) showing the placement of transosseous suture passer with the targeter intraoperatively

A transosseous tunnel was created using a tool with a carrier suture, exiting near the cartilage border (Figure 2).

**Figure 3:**
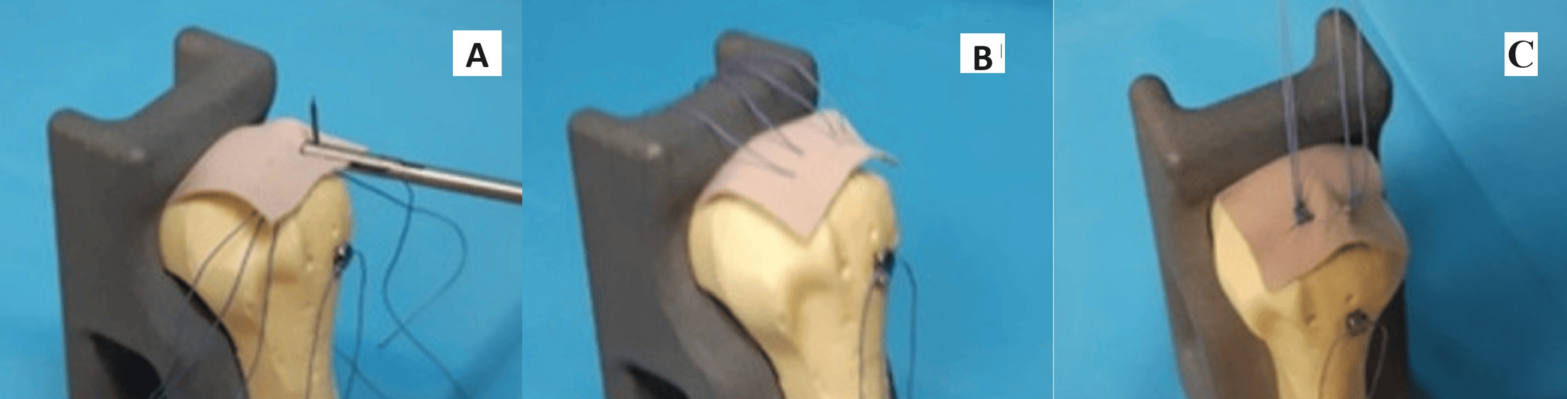
(A) and (B) Passing the sutures through the rotator cuff; (C) tying them in the medial order

Two sutures were loaded onto a transosseous implant, passed through the tunnel with the carrier suture, then pulled through the anterior portal and fixed to the cortex. The sutures from the tunnel were passed through the rotator cuff and tied to create a medial row repair (Figure 3).

**Figure 4:**
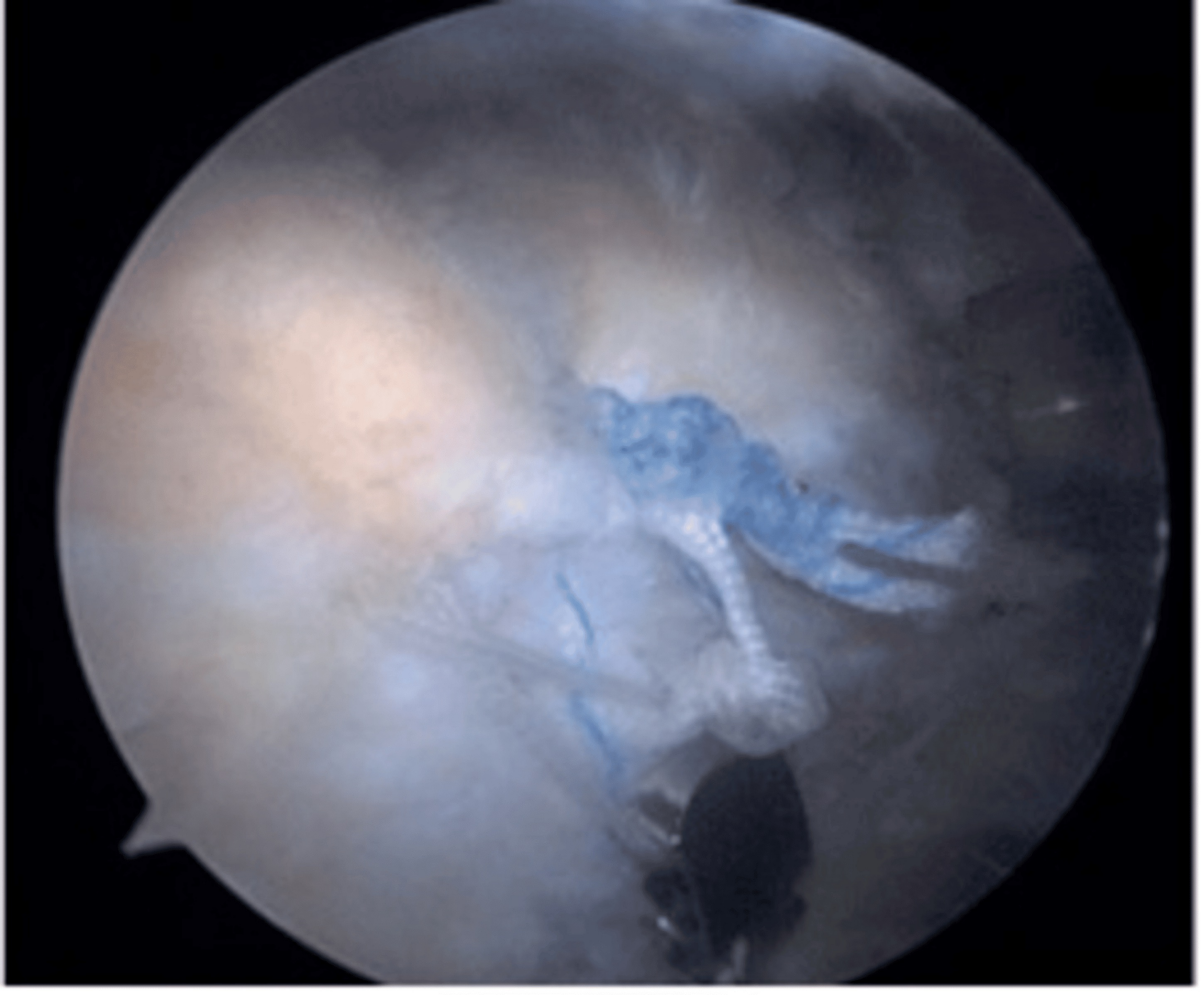
Each suture's end was passed through a hole behind the implant and tied to complete the lateral row repair and suture bridge

Each suture's end was passed through a hole behind the implant and tied to complete the lateral row repair and suture bridge (Figure 4). The coracoacromial ligament was preserved in all patients.

All patients followed a standard rehabilitation program.

Outcome evaluation

**Figure 5:**
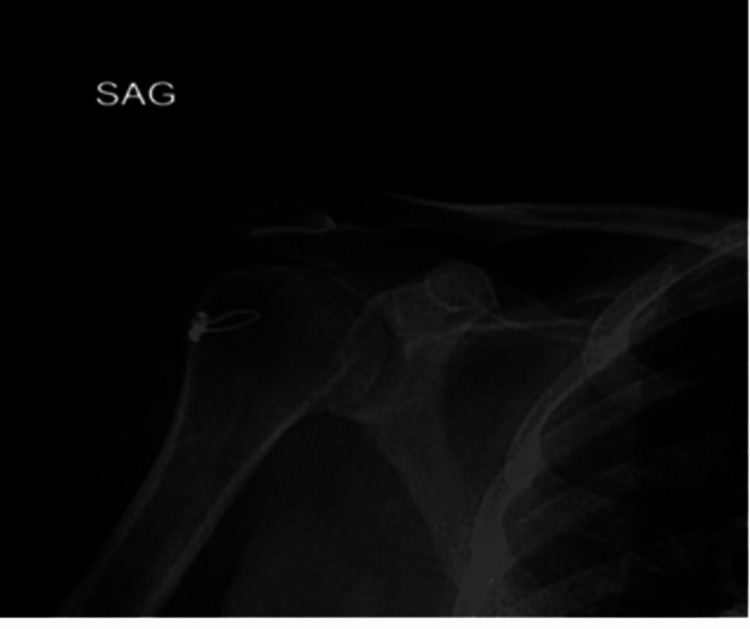
Postoperative antero-posterior shoulder radiograph

Clinical evaluations were performed by an experienced shoulder surgeon. Preoperative and postoperative shoulder X-rays and MRIs were reviewed for tear presence, involved tendons, retraction amount, and fatty degeneration grade (Figure 5).

The shoulder range of motion (ROM) was preoperatively measured with a goniometer. Forward elevation, abduction, internal rotation, and external rotation were assessed at the final follow-up. Tear type was classified according to Ellman and Gartsman [12], and tear size was measured intraoperatively and classified by Bayne and Bateman [13] as small (<1 cm), medium (1-3 cm), large (3-5 cm), and massive (>5 cm).

Clinical outcomes were assessed using Visual Analog Scale (VAS), American Shoulder and Elbow Surgeons (ASES), and Constant shoulder scores preoperatively and at the final follow-up. An experienced musculoskeletal radiologist performed a radiological evaluation, assessing anchor position on standard AP shoulder X-rays and re-tear presence on final follow-up MRIs. According to Sugaya et al. [14], re-tear was considered repair failure and staged.

Statistical analysis

Data were analyzed using SPSS 22.0 (IBM Corp., Armonk, NY). Normal distribution suitability was tested with the Kolmogorov-Smirnov test. Pearson correlation, Kruskal-Wallis, Mann-Whitney U, Pearson chi-square, and Fisher's exact tests were used for various comparisons. Paired samples and Wilcoxon tests evaluated changes over time. A power analysis of 95% was determined, with a significance level set at p<0.05.

## Results

In our study, 33 patients who underwent arthroscopic transosseous surgery with 1-3 cm long tears were evaluated. The mean follow-up period was 26.52±8.14 months. The mean age of patients was 56.0±6.4 years (min: 42, max: 68). 27 patients (81.8%) were female. 32 (97%) of the patients had the right hand dominantly together, and 24 (72.7%) had lesions on the right side. Additional comorbidities were detected in 19 (57.6%) patients, and demographic data are presented in Table 1.

**Table 1:** Demographic data SD: standard deviation.

	Mean ± SD/n(%)
Age	56.0±6.4
Gender, n (%)	
Female	27 (81.8)
Mean	6 (18.2)
Dominant hand, n (%)	
Right	32 (97)
Left	1 (3)
Laterality, n (%)	
Right	24 (72.7)
Left	9 (27.3)
Duration of follow-up (month)	26.52±8.14
Comorbidities, n (%)	
Present	19 (57.6)
Absent	14 (42.4)
Comorbidity subgroups, n (%)	
Hypertension	9 (27.3)
Diabetes mellitus	7 (21.2)
Thyroid diseases	6 (18.2)
Hyperlipidemia	5 (15.2)
Malignity (breast)	1 (3)

The mean tear size was assessed as 22.4±5.9 mm from preoperative MRI data. In addition, the tear shape was determined by examining the surgery reports in the patient files; 19 patients (57.6%) had a crescent-shaped tear, 8 (24.2%) had a U-shaped tear, and 6 (18.2%) had an L-shaped tear (Table 2). Infraspinatus tears were absent in 23 patients (69.7%) and present in 10 (30.3%). Two patients had type 2 SLAP lesions and underwent biceps tenodesis (Table 2).

**Table 2:** Tear size, type, additional pathologies, and procedures performed SD: standard deviation

	Mean ±SD/n (%)
Tear size	22.4±5.9
Tear type, n (%)	
Cresent	19 (57.6%)
U-shaped	6 (18.2%)
L-shaped	8 (24.2%)
Infraspinatus tear, n (%)	
Absent	23 (69.7%)
Present	10 (30.3%)
Type 2 SLAP n (%)	
Present	2 (6%)
Biceps tenodesis, n (%)	
Present	2 (6%)

Our study compared preoperative and postoperative shoulder ROM, pain measurement (VAS), and Constant ASES scores. Significant improvement in active ROM was observed postoperatively (p<0.001), and VAS (3.14±1.20), ASES (88.4±8.12), and Constant (88.9±10.6) scores showed significant differences postoperatively (p<0.05) (Table 3).

**Table 3:** Abduction, forward elevation, internal rotation, external rotation, VAS, ASES, and Constant scores preoperatively and postoperatively VAS: Visual Analogue Scale; ASES: American Shoulder and Elbow Surgeons; SD: standard deviation. *P-values < 0.05 were considered statistically significant.

	Preoperative mean ± SD	Postoperative mean ± SD	p-value
Abduction	84.9±11.1	160.8±9.4	<0.05
Forward elevation	83.6±12.8	159.1±11.7	<0.05
Internal rotation	49.9±3.9	65.3±5.0	<0.05
External rotation	67.1±5.2	83.3±7.7	<0.05
VAS	8.17±1.41	3.14±1.20	<0.05
ASES	46.8 ±15.2	88.4±8.12	<0.05
Constant	32.1±6.4	88.9±10.6	<0.05

Preoperative fatty infiltration was grade 1 in 3 patients (9.1%) and grade 2 in 4 patients (12.1%), with postoperative grades of 9.1% for both. Two patients showed regression in fatty infiltration, though not statistically significant (p>0.05). Seven patients (21.2%) showed repair failure on postoperative MRI (Sugaya III, IV, V). Re-tear patients had significantly higher mean age, larger tear size, and lower postoperative Constant scores (p<0.05) (Table 4).

**Table 4:** Relationship between age, Constant scores, and tear size in rerupture SD: standard deviation. *P-values < 0.05 were considered statistically significant.

	Rerupture		p-value
	Absent (n:26) mean ± SD	Present (n:7) mean ± SD	
Age	54.6±6.1	61.1±4.5	0.012*
Postoperative Constant score	92.7±7.2	75±9.7	<0.05*
Tear size	20.9±5.7	28±1.9	0.002*

One patient developed adhesive capsulitis as a complication, and one had radiological lateral cortex erosion without clinical symptoms due to the flexible implant structure.

## Discussion

The main findings of this study are significant improvements in ROM and Constant shoulder scores and a significant reduction in pain scores following ATO repair. Postoperative pain was absent in 25 patients (75.8%) and mild in 8 (24.2%). Shoulder ROM significantly increased, with average abduction rising from 84.9° to 160.8°.

Many surgical methods for RCT treatment have been compared in the literature, with recent studies showing that transosseous and transosseous equivalent repairs provide the best pressure and strength at the footprint [15-18]. The increased contact area between the tendon and footprint enhances biological healing along with the mechanical strength of the repair. As the footprint surface area in contact with the tendon increases, a stronger healing response is achieved. It has been shown that transosseous sutures provide contact over a broader surface area compared to repair with anchors [10]. Our method combines several repair techniques, offering reliable transosseous fixation, medial and lateral row repair, and transosseous equivalent techniques through implant design, providing a broad contact area and stable repair.

In elderly osteoporotic patients, implant pullout and anchor fixation loss are common causes of RCT repair failure [7]. Revision RCT surgeries often address anchor fixation loss and implant pullout [8]. Anchor fixation strength depends on bone density [9]. Our method ensures solid fixation with a transosseous implant, preventing cortical deformation in osteoporotic bone. By positioning the lateral wings of the implant on the lateral part of the bone, the deformation of the cortex in the osteoporotic bone by the implant was prevented. Since the reliability of the RCT repair technique in osteoporotic bones directly affects postoperative rehabilitation, we were able to confidently implement rehabilitation programs for our patients.

However, our technique has some disadvantages. Sutures from the implant can create dog ears, reducing the tendon-bone contact area. Massive tears require two implants, increasing cost and surgical complexity. The most frequent complication is re-tearing due to insufficient repair [6,19]. Radiological studies report re-tear rates between 29% and 94%, with higher incidences in elderly and massive tear patients [20]. In our study, patients with preoperative grades 1 and 2 fatty degeneration had larger tear sizes and worse repair outcomes, suggesting poor tendon quality in larger tears. Age and RCT size increased re-tear risk, which is consistent with the literature. MRI showed re-tear in 7 of 33 patients (21.2%), with a higher mean age and larger tear size.

Flurin et al. [21] conducted a retrospective study of 576 cases, with a mean follow-up of 18.5 months, and found that patient age influences healing and functional outcomes. However, they concluded that age should not be considered a contraindication for arthroscopic repair. Our study supports their findings, with re-tear patients having a mean age of 61.1 and non-re-tear patients 54.6. Older age correlated with lower postoperative Constant scores. Study limitations include a small sample size, a lack of a comparison group, and a short follow-up period. ATO RCT repair is effective for full-thickness rotator cuff tears, with satisfactory pain alleviation and shoulder function outcomes, as reported by previous studies [22-24]. Our patients’ functional scores at the final follow-up indicate high patient satisfaction with surgical treatment.

## Conclusions

In the repair process, we applied transosseous fixation, medial and lateral row repair, and repaired threads to the tendon, ensuring a wide surface contact and mechanical stability. In this way, we provided easier rehabilitation to our patients. The ATO technique, which provides strong mechanical and biological repair, gave good functional results for full-thickness RCT repair and led to satisfactory healing and clinical results with low complication rates in the short term. However, it is necessary to conduct studies with longer follow-up periods and large series.
